# Towards Computationally Guided Design and Engineering of a *Neisseria meningitidis* Serogroup W Capsule Polymerase with Altered Substrate Specificity

**DOI:** 10.3390/pr9122192

**Published:** 2021-12-06

**Authors:** Subhadra Paudel, James Wachira, Pumtiwitt C. McCarthy

**Affiliations:** 1 Department of Computer Science, Morgan State University, Baltimore, MD 21251, USA; 2 Department of Biology, Morgan State University, Baltimore, MD 21251, USA; 3 Department of Chemistry, Morgan State University, Baltimore, MD 21251, USA

**Keywords:** polysaccharide biomaterials, capsule polymerases, galactosyltransferase, molecular dynamics simulations, bioremediation, protein engineering, *Neisseria meningitidis*

## Abstract

Heavy metal contamination of drinking water is a public health concern that requires the development of more efficient bioremediation techniques. Absorption technologies, including biosorption, provide opportunities for improvements to increase the diversity of target metal ions and overall binding capacity. Microorganisms are a key component in wastewater treatment plants, and they naturally bind metal ions through surface macromolecules but with limited capacity. The long-term goal of this work is to engineer capsule polymerases to synthesize molecules with novel functionalities. In previously published work, we showed that the *Neisseria meningitidis* serogroup W (NmW) galactose–sialic acid (Gal–NeuNAc) heteropolysaccharide binds lead ions effectively, thereby demonstrating the potential for its use in environmental decontamination applications. In this study, computational analysis of the NmW capsule polymerase galactosyltransferase (GT) domain was used to gain insight into how the enzyme could be modified to enable the synthesis of N-acetylgalactosamine–sialic acid (GalNAc–NeuNAc) heteropolysaccharide. Various computational approaches, including molecular modeling with I-TASSER and molecular dynamics (MD) simulations with NAMD, were utilized to identify key amino acid residues in the substrate binding pocket of the GT domain that may be key to conferring UDP-GalNAc specificity. Through these combined strategies and using BshA, a UDP-GlcNAc transferase, as a structural template, several NmW active site residues were identified as mutational targets to accommodate the proposed N-acetyl group in UDP-GalNAc. Thus, a rational approach for potentially conferring new properties to bacterial capsular polysaccharides is demonstrated.

## 1. Introduction

One of the environmental implications of industrialization has been the contamination of water sources with heavy metals either directly (i.e., industrial wastes) or indirectly (i.e., through contaminated soils) [1]. Biosorption, which is the use of microorganisms and/or isolated microbial biomolecules as metal adsorption materials, could provide an environmentally safer route for metal decontamination of water, and several microorganisms and their polysaccharides have been investigated for the ability to bind metals [2,3]. Polysaccharides can be present as a component of the extracellular polymeric substance (EPS) associated with the surface of the bacteria [4]. In addition, some bacteria, particularly Gram-negative bacteria, have capsular polysaccharides that are firmly attached to the outer surface [4,5]. Bacterial capsules are relevant to the understanding of microbial physiology and genetics as well as bacterial host interactions [4,5]. Capsular polysaccharides exhibit great diversity between bacteria in terms of the constituent monosaccharides, glycosidic linkages, and chemical modifications. Thus, they offer a rich source of diverse polysaccharides that could be further developed as biomaterials with varied applications [6]. This diversity underlies the pathogenicity of the Gram-negative bacteria *Neisseria meningitidis* [7], and our focus is on the capsular polysaccharide of serogroup W (NmW) [8,9].

In previous work, the lead-binding affinity of NmW capsular polysaccharides was demonstrated [10]. Solutions of lead were incubated in the absence or presence of NmW polysaccharide (PS) and subsequently passed through a filtration device. The metal solution without NmW PS present was able to pass freely through the filtration device leading to equal concentrations of metal in filtrate and retentate. However, in the presence of PS, less metal was present in the filtrate, indicating binding to the polysaccharide. From these promising results, we have embarked on exploring new ways to broaden the nucleotide-donor substrate specificity of the NmW capsule polymerase to create novel structures with enhanced metal-binding properties.

The NmW capsule polymerase, encoded by the SiaD_W_ gene, is a 120 kDa glycosyltransferase comprised of an N-terminal GT domain, an intervening sequence, and a C-terminal sialyltransferase domain [11–15]. The two catalytic domains differ in the utilization of nucleotide-monosaccharide donors with the GT domain using UDP-Gal and the sialyltransferase domain using CMP-NeuNAc. The N-terminal galactosyltransferase domain is a member of the GT4 CAZy family, while the C-terminal sialyltransferase domain is within the newly classified GT97 CAZy family [13,16]. Mechanistically, GT4 enzymes transfer sugars with retention of stereochemistry at the anomeric carbon and GT97 family members transfer with inversion of stereochemistry at that position. For many of these enzymes, mechanisms are inferred from biochemical and mutational studies with further insight being provided by molecular dynamics simulations [17].

The structures of several members of the BshA family, also classified as GT4 family enzymes, are available. These include family members from pathogenic and non-pathogenic species such as *Bacillus subtilis*, *Bacillus anthracis*, *Staphylococcus epidermis*, and *Staphylococcus aureus* [18–20]. The crystal structures reveal the active site and interactions between the UDP and hexose moieties with specific amino acid residues. These studies revealed the basis for the proposed substrate-assisted reaction mechanism and identified the putative residues involved in the catalytic mechanism [19]. Still, questions remain about the chemical events leading to catalysis.

The studies described here report on sequence analysis, structure prediction, and molecular dynamics of the NmW-GT. Our results have identified several putative ligand binding residues that confer substrate specificity. Simulations and molecular docking could guide the engineering of glycosyltransferases for the synthesis of novel materials.

## 2. Materials and Methods

### 2.1. Sequence Alignments

The genomic sequence of *N. meningitidis* serogroup W capsule polymerase (NmW) (SiaD_W_) was obtained from NCBI [21] (accession No. ABW93688). The full-length sequence is 1037 aa residues long; this work focuses on residues 1–399, which contain the galactosyltransferase domain. The following sequences that encode for β3-GlcNAc-transferases were selected for alignments: LgtA (NCBI accession No. AAL12840), a β3-GlcNAc-transferase from *Neisseria meningitidis* [22–26], and BoGT56a (PDB code 4AYL), an alpha 3-GlcNAc-transferase from *Bacteroides ovatus* [27]. Alignments were performed using BLAST and EMBL-EBI Clustal Omega (ClustalO) [28,29]. All the BLAST parameters were set to default during the BLAST run. The ClustalO multiple sequence alignments were obtained under the default parameters.

### 2.2. Structure Prediction

The galactosyltransferase domain of the NmW capsule polymerase was modeled with I-TASSER [30–33]. The output from I-TASSER provided homology model prediction, ligand binding sites, and active sites. For the subsequent MD simulations and docking studies, the best homology model was chosen based on quality metrics. The best template structure was the glycosyltransferase BshA from *Staphylococcus aureus* complexed with UDP (PDB code: 6D9T) [19]. Its RMSD value was 0.897, with the highest TM-score of 0.76. MolProbity assessment [34–36] was used to determine the model quality. The NmW-GT domain was also modeled with Robetta using the RoseTTAFold option [37,38]. To further validate the quality of models generated from both programs, the structures were submitted to the PROCHECK server [39]. The NmW-GT domain was also submitted to the PSIPRED server for secondary structure prediction [40,41].

### 2.3. Molecular Docking

Docking of UDP-hexoses and the model was conducted with DOCK6 [42–45] as follows: the receptor and ligands were prepared for docking with the Dock Prep program in UCSF-Chimera [46]. Prior to running the Dock Prep program, the crystal structures of UDP-hexose complexes were obtained from the PDB [47]. The protein components were deleted and the ligands were saved separately in PDB format. The Dock Prep step adds hydrogen atoms and charge. The AMBER ff14SB force field was used to add charges. The dot molecular surface (DMS) was generated with UCSF-Chimera using the default settings of 2.0 dots/Å^2^ and probe radius of 1.4 Å.

Spheres were created with sphgen and clusters falling within the proximity of the active site were selected using sphere selector. The default parameters for sphere generation with sphgen were used as follows: spheres were generated to cover the outside surface (R), all points were used (X), the generation of large spheres was suppressed, and maximum and minimum sizes of the spheres were set at 4.0 Å and 1.4 Å, respectively. The location of the active site was estimated firstly by structural alignment of the NmW-GT model with the BshA structure identified by the I-TASSER server in UCSF-Chimera, and a region within 10 Å from the ligand (UDP) was selected with the Zone tool, saved in mol2 format, and used as an input for the sphere selector program. The input for the grid program was generated with Showbox; all spheres in the cluster encompassing the active site were included and the spacing was set at 8 Å.

The Grid program was then used to generate grids using the AMBER_parm99.defn parameters. The docking grid was generated with a spacing of 0.4 Å, and energy scoring was used with the exponent of attractive and repulsive Lennard–Jones terms for VDW set at the default values of 6 and 12, respectively. The coefficient of the dielectric was also set at the default value of 4.0.

Finally, the Dock6.9 program was used to conduct flexible docking on the Stampede2 supercomputer at Texas Advanced Computing Center (TACC), The University of Texas at Austin. Flexible docking searching method was utilized with essentially the default parameters as follows: the minimum number of atoms in an anchor was set to 5; pruning of conformers was used with a maximum of 1000 orientations, 100 clusterheads were retained, and a cutoff of 1.0 was used as the pruning conformer score scaling factor; internal energy scoring was used and the VDW exponent was set to the default value of 12 kcal/mol and the cutoff for pruning conformers was set at 100.0 kcal/mol; minimization of the ligand, anchor, and flexible growth during docking was conducted with the default parameters of 500 iterations, maximum cycles of 1, score convergence of 0.1, cycle convergence of 1.0, translation step size of 1.0, rotation step of 0.1, and torsional step of 10.0.

### 2.4. Molecular Dynamics Simulation

The systems for molecular dynamics simulations were prepared with CHARMM-GUI using the default parameters [48–51]. To simulate the apo-enzyme state, ligand molecules were deleted from the template PDB structure (6D9T) using UCSF-Chimera [46] prior to uploading to the CHARMM-GUI webserver. The systems were prepared essentially using the default parameters. The protein was solvated with TIP3P water and neutralized with potassium chloride [52,53]. A water boundary of 10.0 Å and the KCl concentration of 0.15 M were used in the simulations.

The simulation steps (minimization, equilibration, and production runs) were performed sequentially with NAMD [54,55]. The all-atom molecular dynamics simulations were conducted using default NAMD parameters including Particle Mesh Ewald electrostatics calculations [56]. Equilibration was conducted over six steps as follows: 10 ps of minimization followed by 1.5 ns equilibration steps that were conducted with Langevin dynamics parameters, the default CHARMM36 all-atom additive protein force field [57], and a constant temperature of 303.15 K. Equilibration was conducted with restraints that were gradually reduced over time. Production simulation was conducted for 99 ns in 1 ns blocks each with a 2 fs timestep. The simulations were conducted on XSEDE-PSC-Bridges supercomputer resources [58].

### 2.5. Data Analysis and Visualization

UCSF-Chimera [46], VMD (Visual Molecular Dynamics) [59], and Bio3D [60] were used for MD simulations trajectory analysis. The trajectories were concatenated with CatDCD tool in VMD, and the MD Movie tool in UCSF-Chimera was used to calculate and plot the root mean square deviation (RMSD) and conduct cluster analysis. Root mean square fluctuations (RMSFs), principal component analysis (PCA), and cross-correlation analysis were calculated with Bio3D.

## 3. Results

### 3.1. Sequence Comparison of NmW Capsule Polymerase, LgtA, and 4AYL

**Figure 1.**
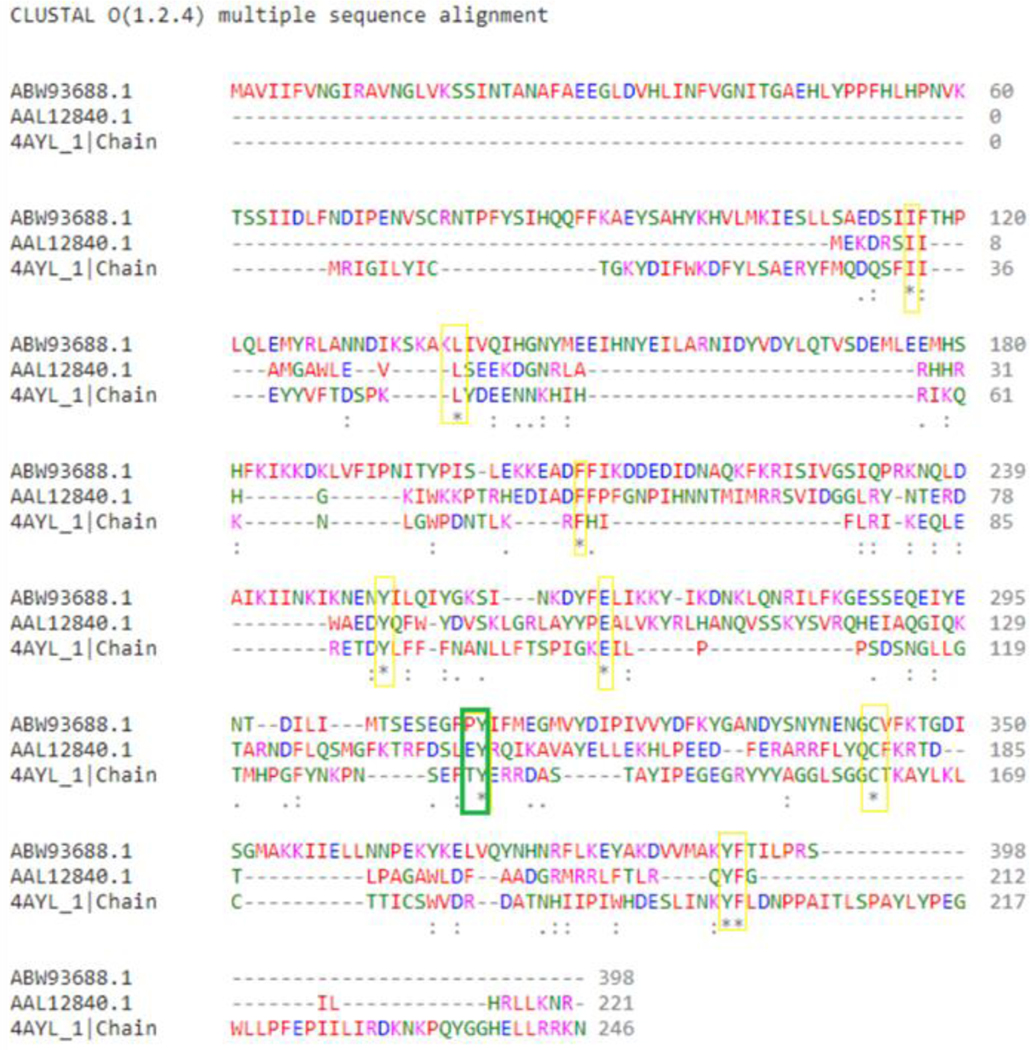
ClustalO multiple sequence alignment of NmW-GT domain (ABW93688.1) aligned with LgtA (AAL12840.1) and BoGT56a (4AYL_1). An asterisk “*” indicates conserved amino acids at that position in all three sequences. A colon “:” represents strong similarity in chemical properties at that amino acid position. A period “.” represents weaker similarity at a particular amino acid position. Identical residues among all three sequences are highlighted by a yellow box. The position of NmW Pro310 is highlighted in green with aligned residues in LgtA and BoGT56a. Reprinted with permission from [63]. Copyright 2021 Subhadra Paudel.

The basis for the substrate specificities of the capsule polymerases from *N. meningiditis* serogroups Y and W towards UDP-Glc and UDP-Gal, respectively, has been mapped to a single amino acid residue in the conserved EX_7_E motif of the GT4 family of glycosyltransferases [11]. Given our goal of engineering specificity for N-acetylgalactosamine, the similarity of the NmW-GT domain with other bacterial GalNAc transferases was investigated. Because of carbohydrate stereochemistry, glycosyltransferases can transfer sugars to an acceptor to create either α or β glycosyl bonds [61]. The NmW capsule polymerase synthesizes α-glycosidic bonds for both GT and ST activities. As such, we focused on two enzymes, LgtA (a β3-GlcNActransferase) and BoGT56a (an α3-GalNActransferase). We performed a sequence comparison of these three sequences using Clustal Omega. There was minimal sequence identity between the three sequences and only nine amino acid residues aligned identically (Figure 1). Previous research has shown that mutation of Pro310 to Gly in the NmW-GT domain can switch specificity from UDP-Gal to UDP-Glu [11]. The alignment results show this proline is aligned with a glutamic acid (Glu) of LgtA and threonine (Thr) of BoGT56a. Interestingly, all three sequences have an identical tyrosine adjacent to this position (Tyr 311 in NmW-GT). The BoGT56a enzyme has a highly conserved Trp189 to Glu192 region which is conserved in all glycosyltransferases of the GT6 family and is known to interact with acceptor substrate in the protein family members for which the three-dimensional structure is known [62]. The hydrophobic property of Trp 189 is conserved in the other two proteins as this position aligns with NmW Tyr 382 and LgtA Phe195. To better visualize areas of conserved sequence, among the NmW-GT domain, LgtA, and BoGT56a, the sequences were input into COBALT. This analysis revealed a conserved region among all three proteins that corresponds to NmW-GT amino acids 235–285 (not shown).

### 3.2. Structure Prediction of NmW-GT Domain and Sequence Alignment with BshA

**Figure 2.**
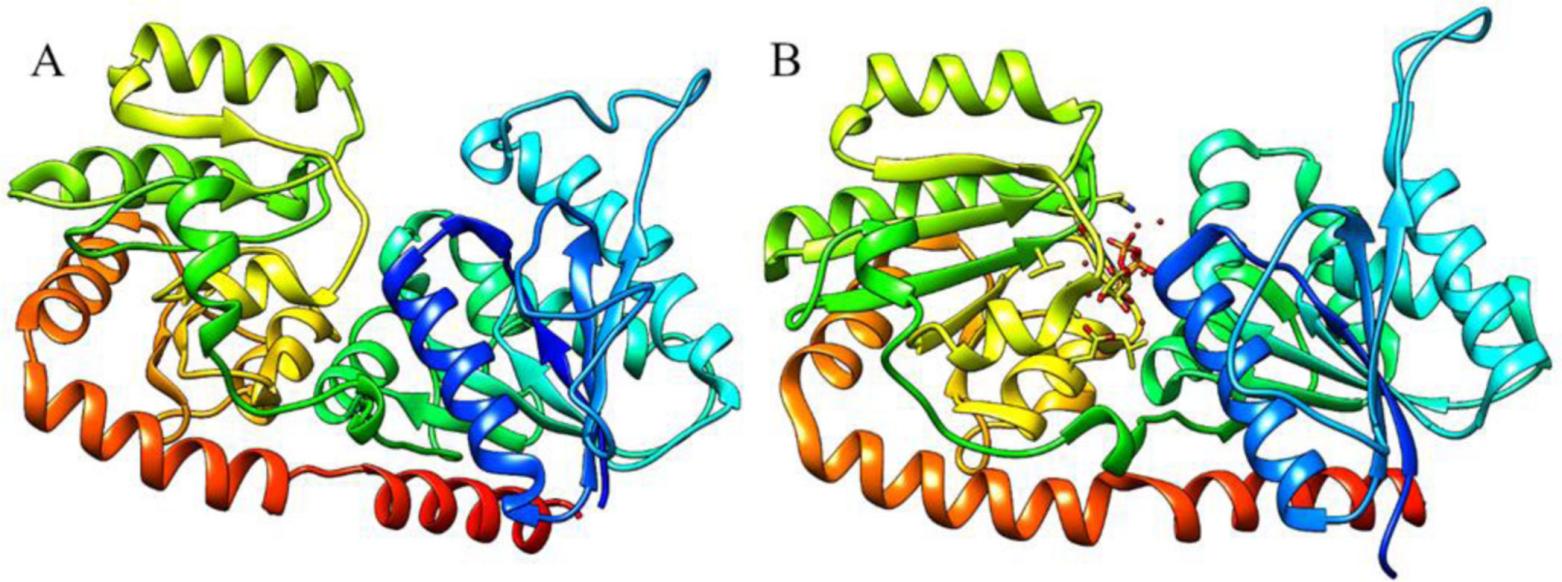
The predicted model of NmW-GT domain. (**A**) I-TASSER-generated model; (**B**) BshA template (PDB: 6D9T). The models are colored from the N-terminus (blue) to the C-terminus (red). The overall topology is considered accurate based on the I-TASSER quality scores [30,32,70].

In the absence of a crystal structure of the NmW capsule polymerases, structure prediction of the NmW-GT domain was performed using I-TASSER [31–33]. To arrive at the structural models, the I-TASSER program uses a rigorous, multistep approach using LOMETS [64,65]. LOMETS is a metaserver comprising multiple threading programs, and the final I-TASSER-predicted models are derived from the top 10 scoring templates obtained from LOMETS. For the NmW-GT domain, the top 10 templates were from five distinct proteins. A UDP-GlcNActransferase from *Staphlyoccocus aureus*, BshA (PDB ID: 6D9T), was the top structure with the highest C-score of 0.31 (a measure of confidence in the model) and shared 17% sequence identity with the NmW-GT domain. This protein plays a functional role in bacillithiol biosynthesis. Both enzymes are members of the GT4 CAZy family (Figure 2). The four additional proteins were MshA, a UDP-GlcNActransferase from *Cornybacterium glutamicum* involved in mycothiol biosynthesis [66] (PDB ID: 3C4Q with 14% sequence identity with NmW-GT domain); the catalytic domain from starch synthase IV, an ADP-Glctransferase from *Arabidopsis thaliana* [67] (PDB ID: 6GNE and 16% sequence identity); a GT4-family glycosyltransferase of unknown function from *Bacillus anthracis* [68] (PDB ID: 2JJM and 19% sequence identity); and TarM, a UDP-GlcNActransferase from *Staphylococcus aureus* involved in the production of teichoic acid found in the bacterial cell wall [69] (PDB ID: X7P and 19% sequence identity).

**Figure 3.**
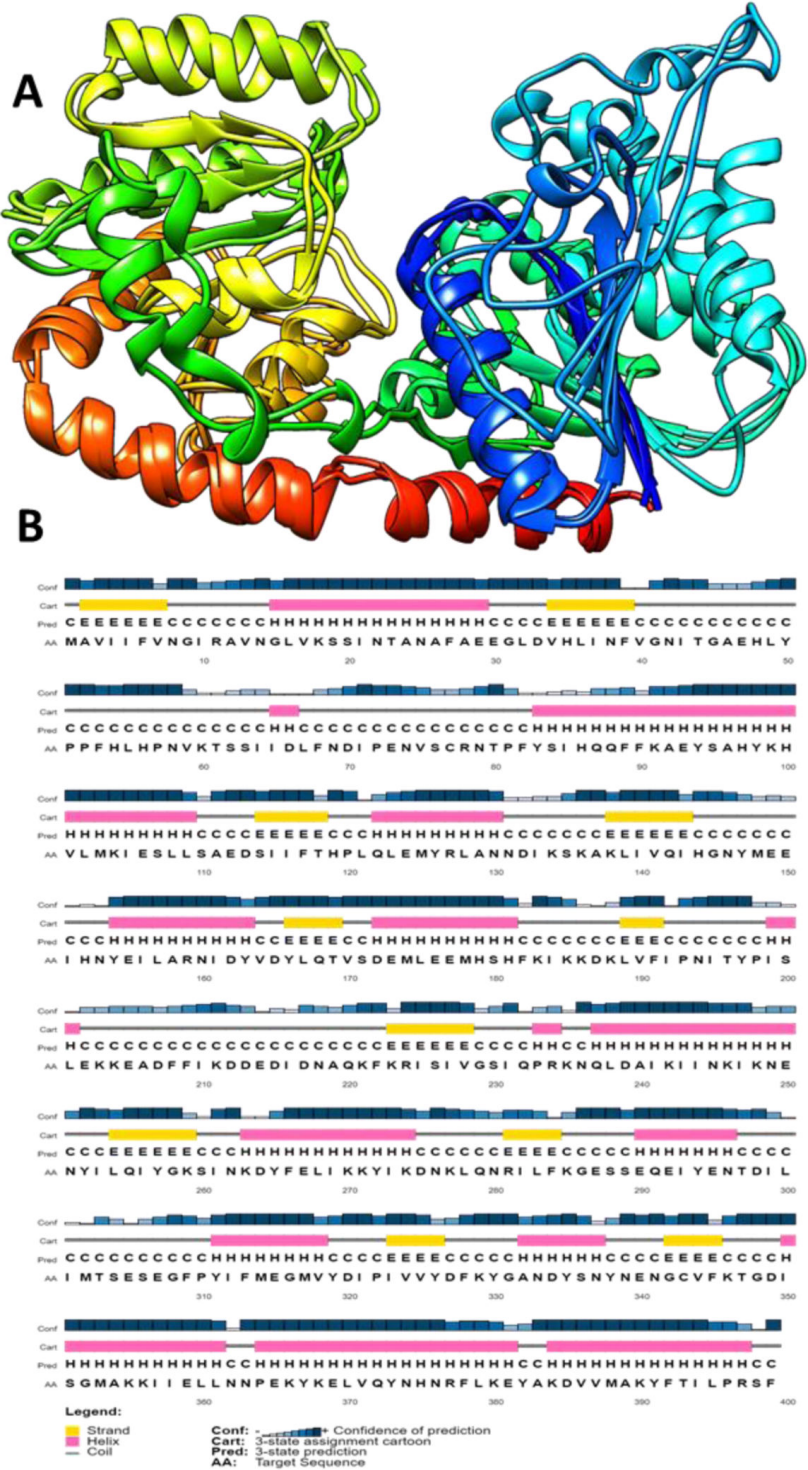
Comparison of NmW-GT domain structures predicted by alternative programs. (**A**) The I-TASSER model was overlaid with Matchmaker on the model predicted with Robetta. The confidence of prediction by Robetta is 0.7270. (**B**) Secondary structure prediction with PSIPRED gave results consistent with the 3D structure prediction methods used.

In addition to homology modeling with I-TASSER, the NmW-GT domain was modeled with Robetta using the RoseTTAFold option [37,38]. Robetta is based on RosettaCM, which combines homology and de novo modeling with structure refinement [71]. The program has incorporated additional neural networks based on three levels of protein structure, and it produces structures with high accuracies [37]. Comparison of the two models was performed with TM-align webserver [70], and the results show a high level of agreement with an RMSD of 2.87 and TM-score of 0.87 (Figure 3A). PROCHECK was conducted on the models, and the Robetta model had favorable scores for all parameters except side chain parameters and eight residues comprising 2.2% of the total that were in disallowed regions of the Ramachandran plot. The I-TASSER-predicted model had six residues in disallowed regions, and errors were identified in the other criteria as well. Nevertheless, the model was considered adequate for defining the active site given the fold accuracy estimated by both structure prediction programs. The TM-score ranges from 0 to 1; a score of 0.3 or below indicates random structure similarity. PSIPRED secondary structure prediction correlates well with both models [40,41] (Figure 3B). The predicted model structure was used to investigate potential interactions with UDP-hexose substrates.

The different poses of UDP-hexoses based on crystal structures are shown in Supplementary Figure S1. The rotatable bonds notwithstanding, the differences in the hexose moieties of interest are located at two carbon atoms: C2, which carries an -OH or N-acetyl group, and C4, where the difference is in the orientation of the -OH group. Analysis of protein–ligand interactions was based on the crystal structures of BshA bound to UDP and with UDP and N-acetylglucosamine (GlcNAc) as reported in the literature [19]. NmW-GT differs in substrate specificity in that it utilizes UDP-Gal, and these studies seek to change the specificity of this domain to UDP-GalNAc. In further analysis, we highlight a series of functionally relevant residues that can inform our studies to alter the specificity of the NmW-GT domain.

**Figure 4.**
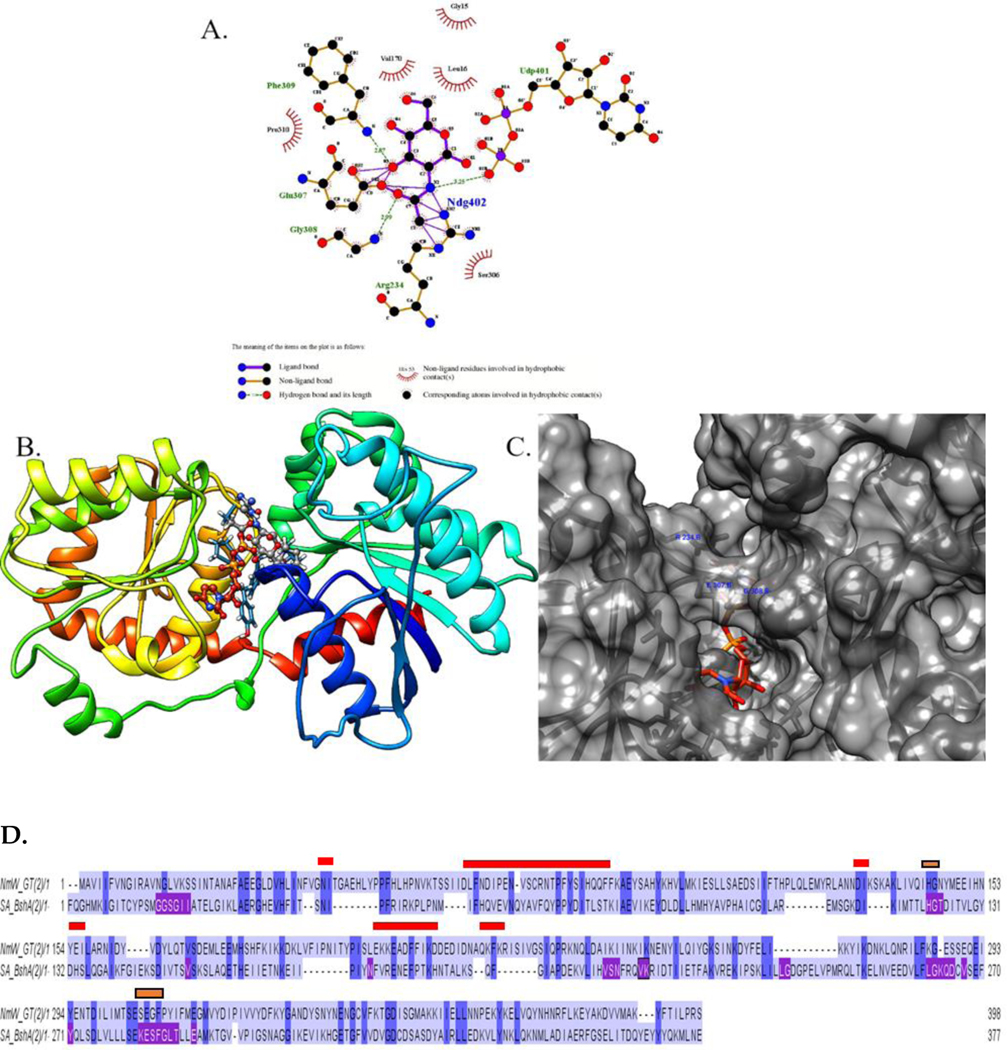
Model of the NmW-GT active site residues. (**A**) Proposed interactions of NmW-GT with UDP-N-acetylglucosamine. A 2D interaction map was generated with LigPlus and the legend is also shown. (**B**,**C**) Location of the active site identified through structural alignment. The NmW-GT model and BshA crystal structure were aligned with Matchmaker in UCSF-Chimera, and then BshA protein sequence was deleted. The UDP-GlcNAc ligand is colored using CPK scheme, and some amino acid side chains in the active site are shown as blue bonds on the ribbons. Residues potentially interacting with N-acetyl moiety and 4-OH groups are labeled in (**C**). (**D**) Pairwise sequence alignment of NmW-GT and BshA. Red indicates highly fluctuating regions in the molecular dynamics simulations (Supplementary Figure S3), purple indicates the residues in BshA that interact with the ligand, and gold represents residues that interact with the GlcNAc moiety in BshA and are conserved in NmW-GT. Secondly, docking was conducted with DOCK6, and the results are summarized in Supplementary Figure S2.

Based on the structure of BshA complexed with UDP and N-acetylglucosamine, a model of NmW-GT in complex with the natural ligand UDP-galactose and with UDP-N-acetylgalactosamine was generated within UCSF-Chimera. Through structural alignment in UCSF-Chimera and Matchmaker, N-acetylglucosamine was placed within the putative NmW-GT active site, and the residues within 3 angstroms of the ligand were identified as L16, H144, V170, R234, and the motif EGFPY 307–311. Among these nine residues, four are identical between the two proteins (H144, V170, E307, and F309) (Figure 4). It has been shown in other published studies that mutation of NmW-GT E307A leads to the complete abolishment of galactosyltransferase activity in that domain [19]. In addition to the likely effects due to the stereochemical differences of glucose and galactose moieties at carbon 4, the residues in proximity to the N-acetyl group (R234 and S306) are not conserved between the two proteins and are good targets for modification. The 2D representations of putative ligand–protein interactions were generated with LigPlot+ [72].

**Figure 5.**
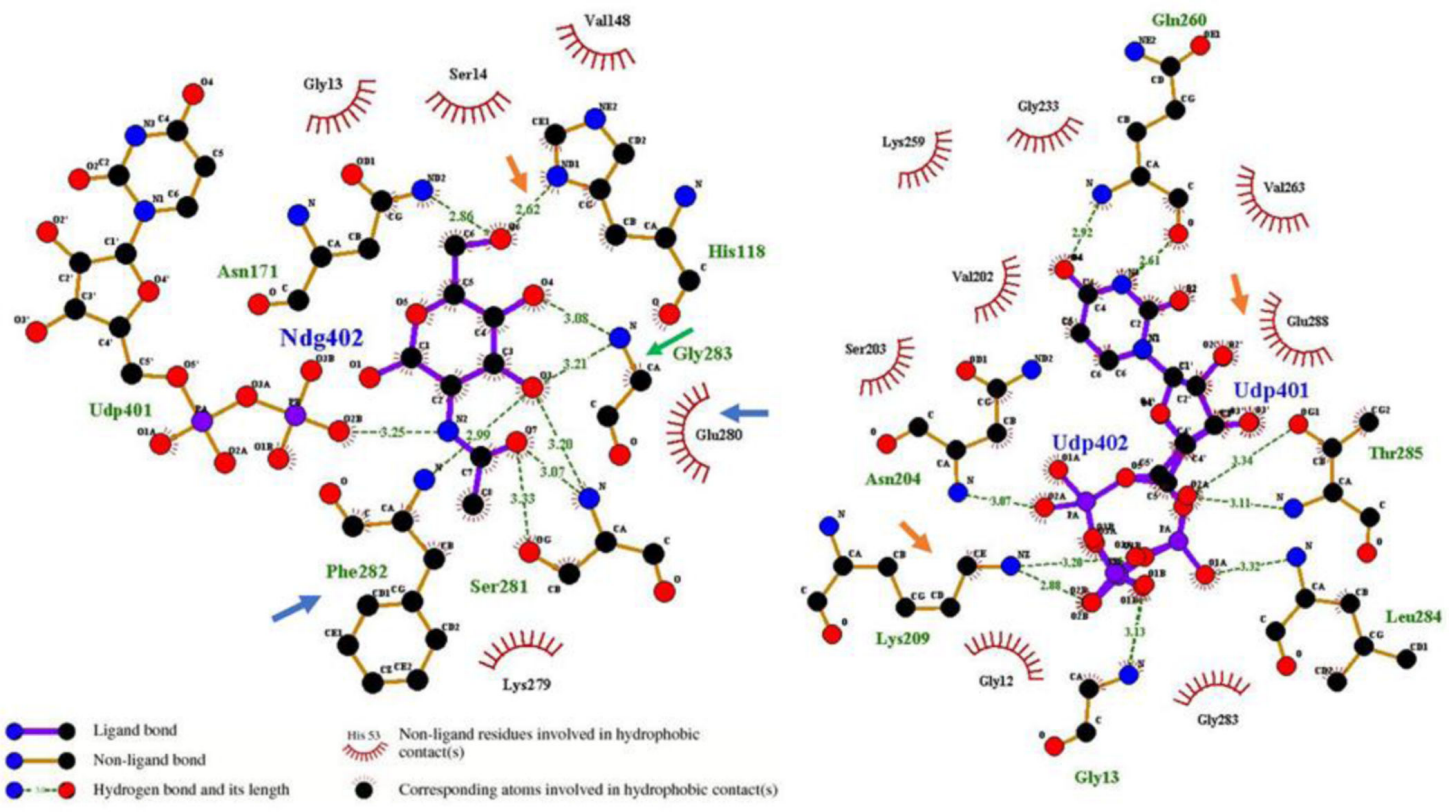
Putative ligand binding residues in NmW-GT based on the modeling template. Ligand-proximal residues in the template structures were identified with LigPlot+. Arrows point to the ligand-proximal residues that are conserved in BshA and NmW-GT.

The NmW-GT domain was aligned to selected BshA sequences to better map functional motifs as identified through X-ray crystallography of BshA. In panel C, residues highlighted in purple are determined to be within 5 angstroms of the ligands (BshA sequence), and they map to the same regions identified through experimental data by other authors. The region containing the ESFG motif (280–283; numbering is based on *S. aureus* BshA) that is identified as forming hydrogen bonds with GlcNAc (Ndg402) in *S. aureus* BshA is highly conserved in BshA enzymes. Two residues that interact directly with GlcNAc in this motif (E and F) are also conserved in NmW-GT (see the gold paneling in Figures 4D and 5). Of special note is that the G is replaced with P in NmW-GT. Mutation of this residue to G in NmW-GT changes the enzyme’s specificity for UDP-Gal in favor of UDP-Glu [11]. Other residues that form hydrogen bonds with GlcNAc in BshA are H118 and N171. H118 is conserved in NmW-GT but N171 is not (Figure 4C). Whereas in the crystal structure this residue interacts with the C6 O and not the anomeric carbon (see the gold arrow in Figure 5), mutational data suggest a critical role in catalysis. Other functionally important residues are T120, which is replaced with N in NmW-GT; K209 and E288, which are conserved in NmW-GT; and E280, which is also conserved in NmW-GT (see the blue arrow in the left panel). E280 and E288 align with E307 and E314 of the NmW-GT. These residues are part of a conserved EX_7_E motif known to be critical for nucleotide donor sugar recognition in GT4 family members [73].

While the manual docking was useful in locating the NmW-GT active site, further evidence was obtained through software-guided docking. DOCK6 was used to predict the interaction of NmW-GT with UDP-hexoses. Of note, DOCK6 placed UDP-GalNAc in a similar orientation to that reported for the template (Supplementary Figure S2). A superposition of the BshA–ligand complex and the docked NmW-GT–UDP-GalNAc places the UDP groups within 1.63 angstroms of one another; however, there is a displacement of the sugar moiety by 14 angstroms, which can be attributed to single bond rotations.

**Figure 6.**
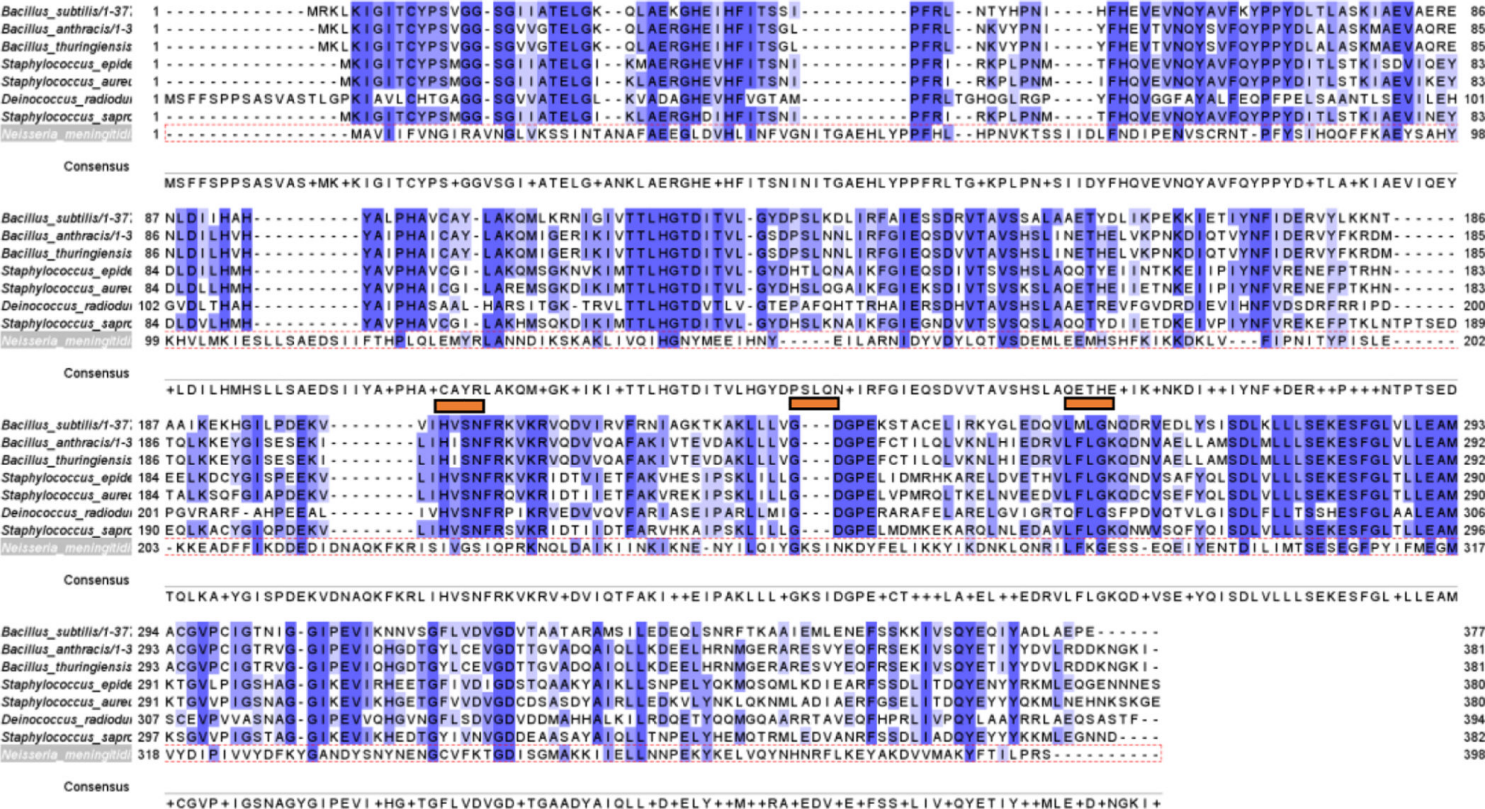
Comparison of NmW-GT with BshA sequences from different species. Blue highlights regions of conservation, and the gold bars highlight regions that interact with the substrate in BshA enzymes and that are not conserved in NmW-GT.

**Figure 7.**
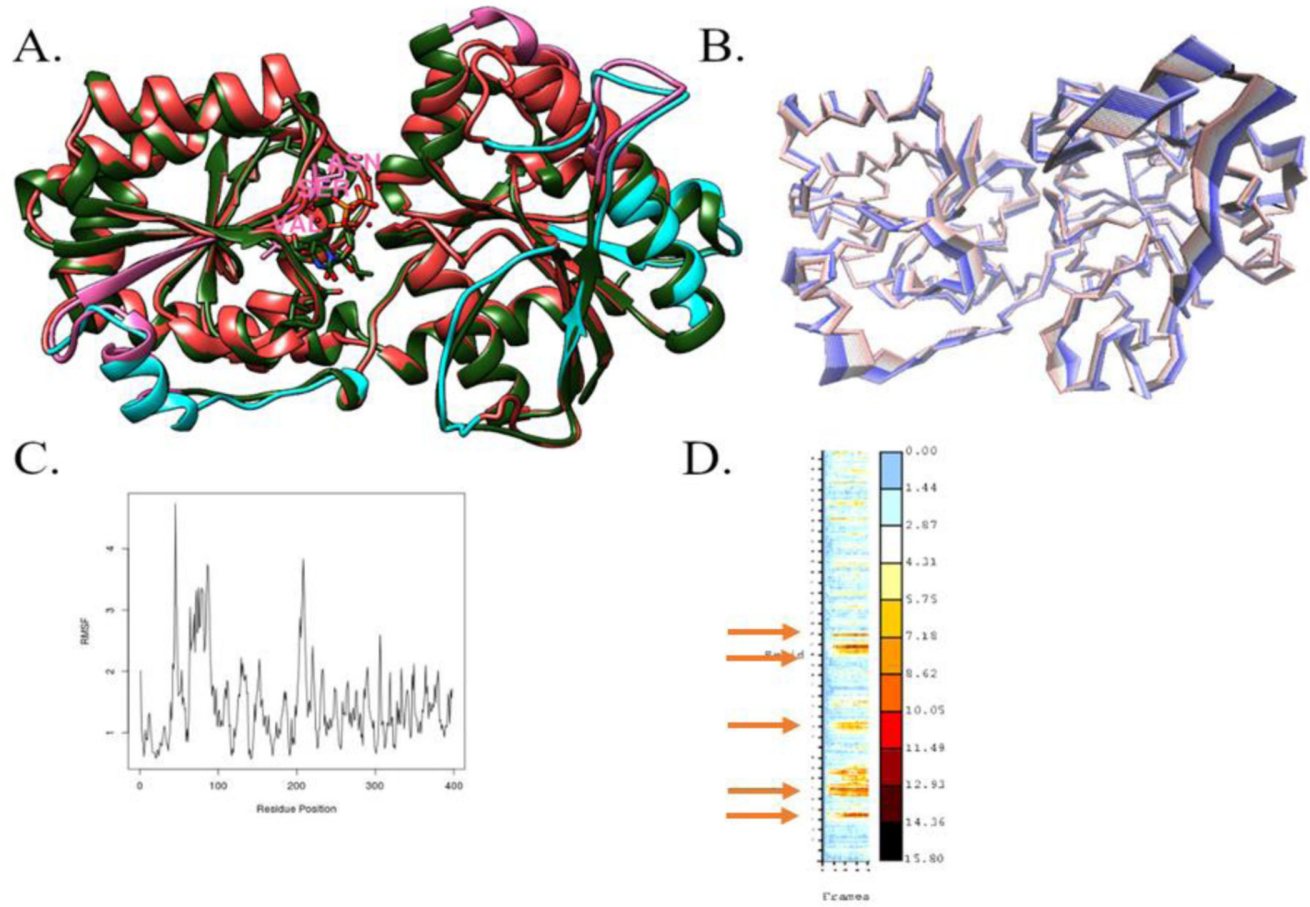
NmW-GT conformation changes. (**A**) RMSF analysis was conducted on MD trajectories and the regions showing highest conformational changes were highlighted with UCSF-Chimera in pink (BshA) and cyan (NmW-GT). The template (PDB ID: 6D9T) and the NmW-GT model were aligned with Matchmaker in UCSF-Chimera. (**B**) Segments with the highest flexibility in NmW-GT as revealed through principal component analysis (PCA). (**C**) Plot of the contributions of each residue to fluctuations of the NmW-GT. (**D**) Heatmap of the RMSD. The regions experiencing the highest mobility in NmW-GT are as follows: residues 42–48, 64–91, 128–136, 154–156, 201–214, and 219–223; gold arrows, also see Supplementary Figure S3.

While a discrepancy is noted in the manually docked and DOCK6-generated poses, it is noted that the positioning of the UDP section of the ligand is similar in both cases and with the crystal structure of the template proteins. The most energetically favorable cluster lies proximal to the active site (Supplementary Figure S2B). Another cluster of conformers overlaps with the UDP binding site, but the hexose moiety is positioned in a different cavity (Supplementary Figure S2C). Hence, these studies create a starting point for advancing the structural biology of this class of proteins. The docked structure was equilibrated and subjected to a short (20 ns) simulation which revealed stability of the ligand in the active site. A multiple sequence alignment was conducted with selected BshA orthologs and with NmW-GT (Figure 6), and data show the high conservation of these functionally important residues. Still, several substrate-proximal residues in BshA are not conserved in NmW-GT (see the gold bar in Figure 6). Structural alignment with Matchmaker [74] reveals the potential positioning of the substrate binding residues and a good agreement of the model with the template, with an RMSD of 0.540 angstroms between 265 pruned atom pairs or 3.586 across all 363 pairs (Figure 7A).

### 3.3. Molecular Dynamics Simulations

The NmW-GT domain model used in these studies was generated with I-TASSER [30–33] using the *Staphylococcus aureus* bacillithiol biosynthesis glycosyltransferase (BshA) as the template. The structure of *S. aureus* BshA was solved in the presence of UDP and in the presence of UDP and N-acetylglucosamine (GlcNAc) [19]. The structure revealed substrate binding residues and the involvement of the substrate in the catalytic mechanism. Molecular dynamics simulations were conducted to further investigate the conformational spaces that are accessible to the critical residues [19]. Among the highly flexible regions, the Val-Ser-Asn (202–204) segment of BshA is near the substrate (Figure 7, residues rendered in stick format and highlighted in pink and Supplementary Figure S3). K209, which is next to this region, is implicated in catalytic activity and, whereas the VSN sequence is not conserved in NmW-GT, K is conserved (Figure 4D). The flexible regions are highlighted in pink in BshA and in cyan in NmW-GT, and partial overlaps are apparent (Figure 7A) and are visualized through principal component analysis for NmW-GT (Figure 7B). Regions contributing the most to the structural fluctuations are primarily in the terminal regions, as expected, the periphery of the structure, but also at the interface of the two domains, which is also proximal to the active site.

Equilibration of the system was confirmed via RMSD analysis within UCSF-Chimera (Supplementary Figure S4A). Further, network analysis of the trajectory with structure-Viz [75] predicts additional residues that are not proximal to the active site but are predicted to interact with the residues of interest (Supplementary Figure S4B), and cluster analysis indicates relative lateral and rotational movements between the domains (Supplementary Figure S4C).

#### Cross-Correlation of Fluctuating Residues

**Figure 8.**
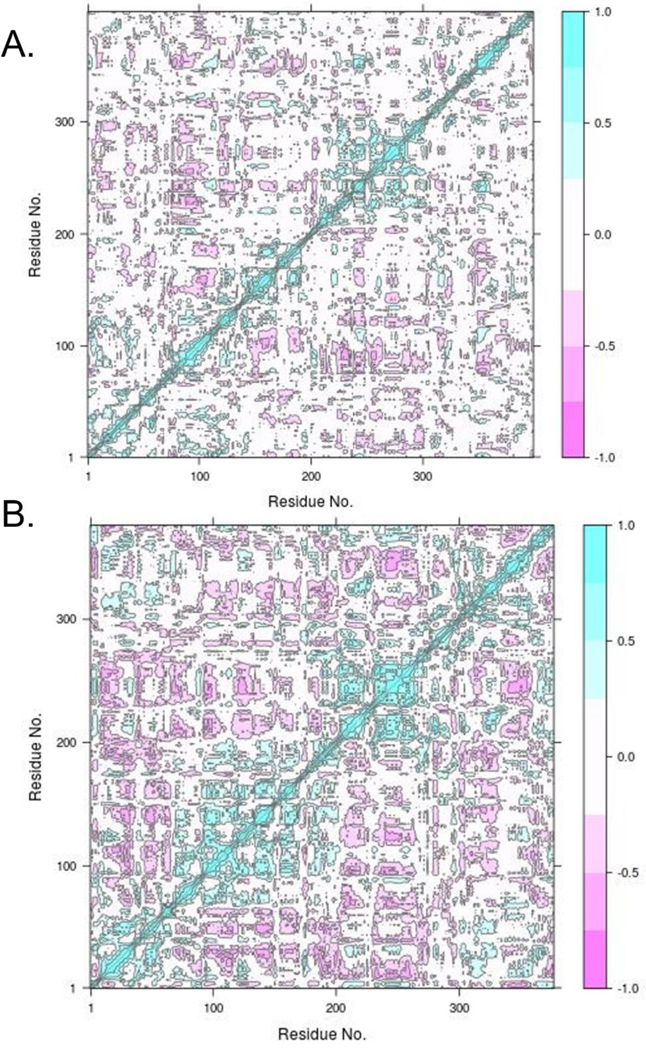
NmW-GT correlative motions. (**A**) NmW-GT model; (**B**) BshA structure. The DCCM matrix was constructed and plotted with Bio3D R library. Regions that move in a correlated manner are shown in green and anticorrelated ones are shown in pink. Reprinted with permission from From [63]. Copyright 2021 Subhadra Paudel.

A dynamic cross-correlation matrix (DCCM) was constructed to determine the regions that move in concert during the trajectory (Figure 8). Incidentally, the regions with high degrees of fluctuation appear to be correlated in the NmW-GT model (green in Figure 8A). The DCCM statistics of the template structure (6D9T) (Figure 8B) highlighted the following regions as positively correlated during MD simulation: aa 21–90, which starts with α-helix structure and ends at a loop region; aa 207–328, which begins and ends with α-helix structure with a mixture of structures in between; and aa 356–376, which falls under α-helix structure.

## 4. Discussion

Heavy metal environmental pollution remains a challenging problem that could potentially be alleviated through biosorption. Microbial polysaccharides have been shown to effectively bind lead in water. To enhance chelation and hence the capacity for bioremediation, biosynthetic routes for capsular polysaccharides with desired properties are currently under development. The GT domain of the *Neisseria meningiditis* serogroup W capsule polymerase was modeled to inform experimental studies that seek to alter the substrate specificity of this domain. We identified putative active site residues through structure prediction approaches that incorporate de novo and homology modeling and sequence alignments. Molecular dynamics simulations also revealed regions of fluctuation that surround the active site region and that might influence enzyme conformations and hence activity.

In the context of the full-length NmW capsule polymerase, others have shown that P310G mutation leads to a switch in nucleotide donor specificity from UDP-Gal to UDP-Glu [11]. This can be interpreted as an indication that, like BshA, the Gly then forms hydrogen bonding interactions with the C3 and C4 OH groups of glucose (Figure 5). Given that the main chain NH group that forms the hydrogen bond is lacking in proline, it is tempting to speculate that the proline and the subsequent tyrosine (Y311) enable interactions that are favorable to specificity for galactose. S281 forms H-binding interactions with the acetyl moiety of GlcNAc in BshA (Figure 5), and this residue is replaced with a G in NmW-GT. Thus, this region merits further investigation. While our results await experimental validation, others have reported that the alteration of a single amino acid in β1,4-galactosyltransferase biases substrate utilization from UDP-Gal to UDP-GalNAc [76]. Further, conformational changes mediated by protein–protein interactions were important in determining substrate specificity [76].

Mutation of either H118, T120, K209, E280, or E288 led to the complete loss of BshA catalytic activity, suggesting a role for these residues in the reaction mechanism [19]. This is consistent with other studies that reported that the highly conserved EX_7_E motif of the GT4 family mediates catalysis. Still, while E280 is proximal to the reactive bond, E288 is positioned closer to the uracil group, which is consistent with the observation that the mutation of the initial E in the AceA α-mannosyltransferase from *Acetobacter xylinum*, also a GT4 enzyme, leads to a complete loss of enzymatic activity while mutants of the second E retain some residual activity [77]. This motif is conserved in NmW-GT but several ligand binding residues exhibit lower levels of conservation. K209 was identified as playing the catalytic role of interacting with a β-phosphate in the UDP-sugar moiety [19]. MD simulations determined that the main chain amide group of G13 and the side chain of K209 are proximal to the β-phosphoryl group of the UDP moiety and hence could mediate the displacement of the leaving group [19]. In this study, structural alignments show the conservation of these residues in NmW-GT. There are three types of mechanisms proposed for retaining glycosyltransferases: SN_2_, double displacement, and substrate-assisted SN_i_. Recent crystallography and enzymatic studies have definitively ruled out two of the three. H118 was shown to play a critical role in stabilizing the oxocarbenium-like intermediate that develops by the SN_i_-like mechanism. Although the NmW-GT domain has been characterized as a retaining glycosyltransferase, the mechanistic details of how this transfer takes place have not been determined due to the lack of a three-dimensional structure. Given that H118 and the aforementioned residues except T120 are conserved in NmW-GT, the present work alludes to a similar catalytic mechanism for this domain.

Some BshA mutations have been reported to eliminate enzymatic activity despite not being proximal to the substrate reacting centers [19]. The results of MD simulations identified additional residues in the regions between the substrate binding residues that could contribute to conformational changes that mediate activity (Figure 6 and Supplementary Figure S3). In closing, these studies have utilized a combined strategy of sequence alignment, structure prediction, and MD simulations to provide new insight on potential active site residues of the NmW-GT that can be modified to engineer new nucleotide-donor substrate specificity. Future work will target these residues for mutation and assess the activity of the NmW capsule polymerase to transfer GalNAc to an acceptor.

## Supplementary Material

Supplementary Material

## Data Availability

The data from this work are available upon reasonable request from the authors.
