# Supplementary material for: Towards Computationally Guided Design and Engineering of a Neisseria meningitidis Serogroup W Capsule Polymerase with Altered Substrate Specificity
Source: Processes (Basel). Author manuscript; Available in PMC 2023 Jul 21. (PMC10361700; doi:10.3390/pr9122192)
Supplement: Supplementary Material [file NIHMS1900607-supplement-Supplementary_Material.pdf]

Supplementary Figures

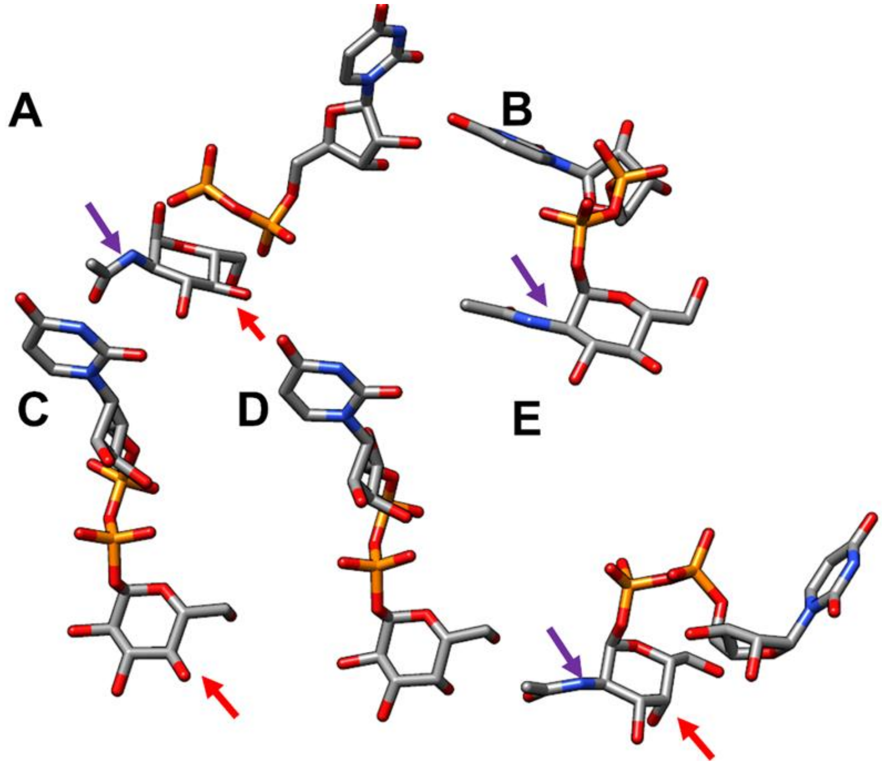

Supplementary Figure S1. Selected molecular structures of UDP-sugars as enzyme substrates. The figure highlights chemical differences rather than conformations. The purple arrows highlight the position of the N-acetyl moiety on C2 and the red arrows highlight the position of the -OH group on C4 whose orientation differs between glucose or galactose. A) UDP and N-acetylglucosamine from a co-crystal structure with *Staphylococcus aureus* BshA (PDB ID: 6N1X) [1]; B) UDP-N-acetylglucosamine from co-crystal structure with *E. coli* N-acetylglucosamine 1-phosphate uridyltransferase (PDB ID: 1FWY) [2]; C) The structure of UDP-glucose from a complex with galactose-1-phosphate uridylyltransferase (PDB ID: 1GUQ) [3]; D) UDP-galactose structure from a complex with galactose-1-phosphate uridylyltransferase (PDB ID: 1GUP) [3]; and E) the structure of UDP-N-acetyl-galactosamine from a complex with bovine Beta1,4- Galactosyltransferase (PDB ID: 1OQM) [4]. The structures were obtained from the PDB and protein components deleted with UCSF-Chimera [5].

A.

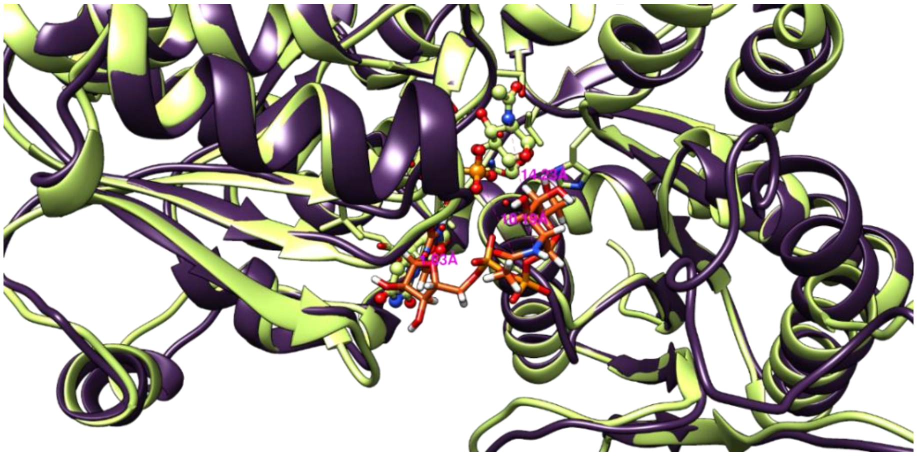

B

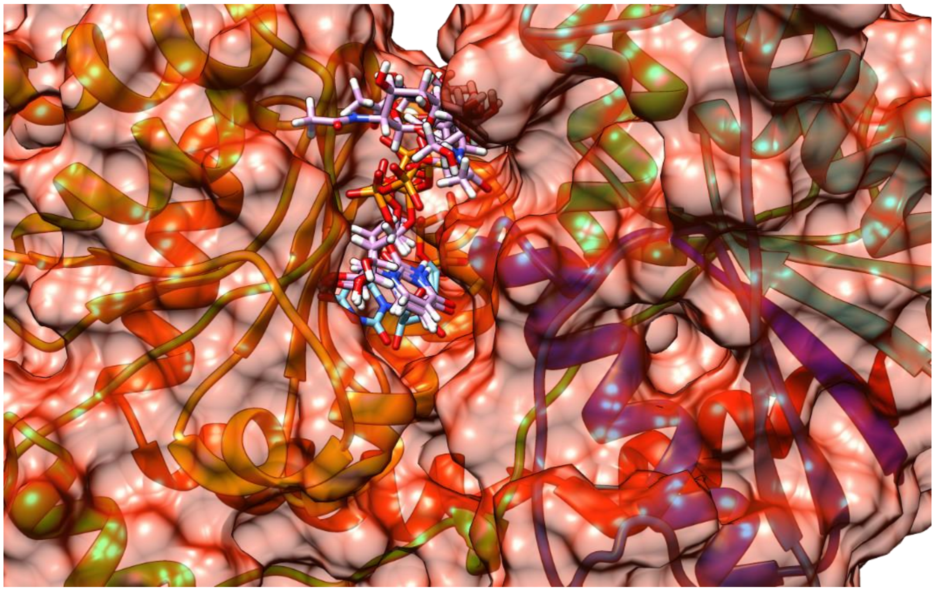

C.

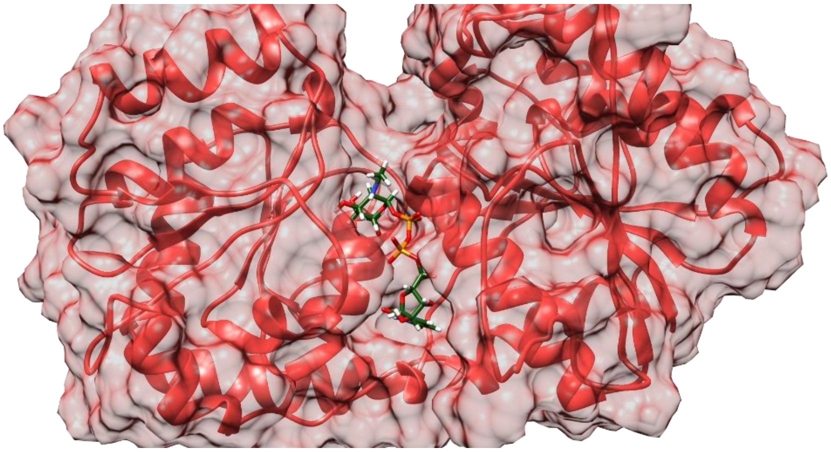

Supplementary Figure S2. Docking of GalNAc to the NmW-GT model. A) Coordinates for UDP-GalNAc were obtained from the PDB ID:1OQM [4]. After docking, BshA structure was aligned with the NmW-GT complex in UCSF-Chimera. BshA enzyme is shown in green and its ligand in ball and stick rendering. B) and C) Docking was repeated as in A but several conformers were generated. Again, the UDP moieties of BshA and NmW-GT align closely in the most stable conformers of the latter, but the sugar moiety is offset from the proposed active site. In C) the ligand is in alternative cavity that overlaps with the putative active site. A total of 50 conformers were generated and examined.

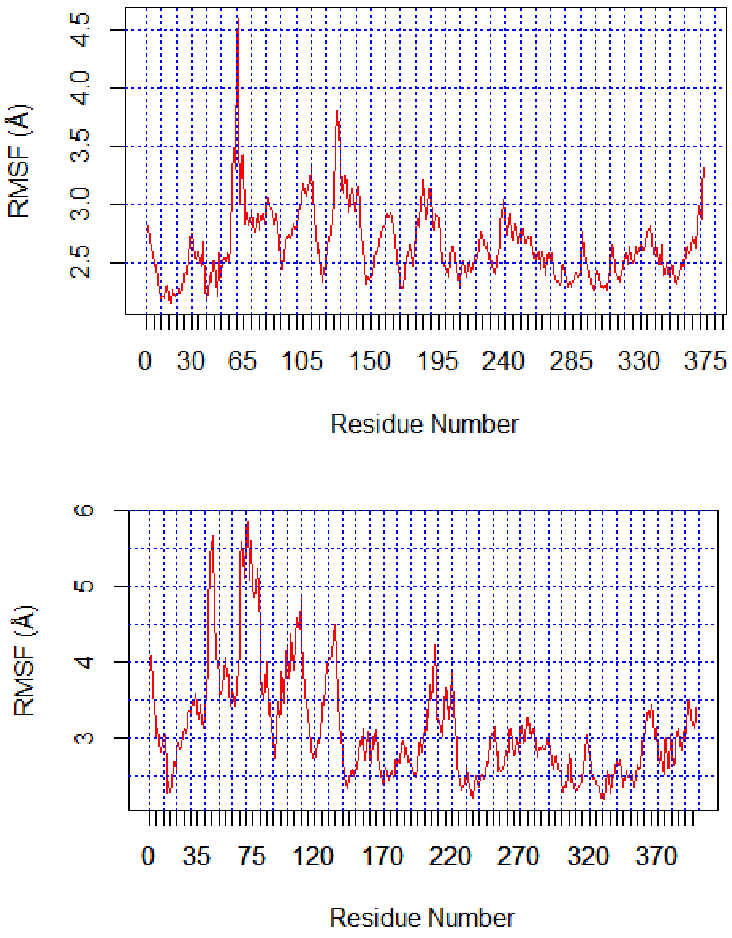

Supplementary Figure S3. Residue-wise mobility during MD simulation. <u>Top panel</u>: *S. aureus* Bsha. Regions with the highest fluctuations are 55–65 (NQYAVFQYPPYD) and 125-135 (LQGAIKFGDHS). <u>Bottom panel</u>: NmW-GT domain. Highest fluctuations are observed in regions 40–50 (VGNITGAEHLY), 6080 (KTSSIIDLFNDIPENVSCRNT), 100110 (HVLMKIEESLLLS), 130-140(NNDIKSKAKLI), and 200–220(SLEKKEADFFIKDDEDIDNAQ). The vertical gridlines are spaced by 10 residues and tick marks by 5 residues. The top panel combined trajectories at the first 10 ns, 35 to 45 ns and the last 10 ns of the total 99 ns trajectory. The bottom panel combines the initial 10 ns and the last 10 ns of the trajectory.

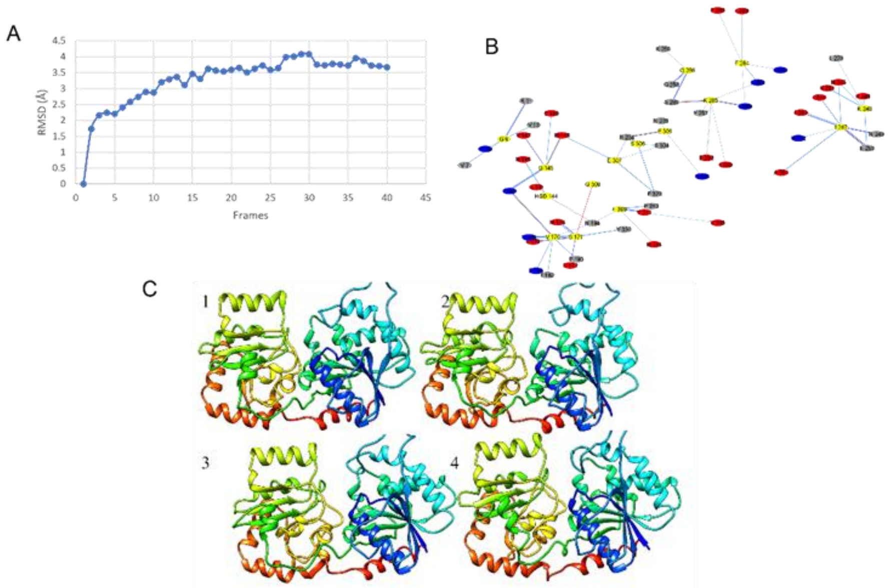

Supplementary Figure S4. MD analysis of the NmW-GT model. A) Frame RMSD analysis, B) Residue interaction network, and C) Representative clusters of the trajectory. NAMD trajectory was analyzed to identify residues with coordinate movements as well as larger-scale domain movements. The residues are colored as follows in panel B: yellow are the selected residues based on predicted proximity to the ligand, red are residues within helices, blue are residues within beta sheets and in gray are residues located within loops. The simulation was conducted in 10 ns blocks based on the configuration and MD simulation scripts generated by CHARMM-GUI. The frames analyzed are for blocks 1, 10, 35 and 99 and encompass the full trajectory.

1. Royer, C.J.; Cook, P.D. A structural and functional analysis of the glycosyltransferase bsha from staphylococcus aureus: Insights into the reaction mechanism and regulation of bacillithiol production. *Protein Science: A Publication of the Protein Society* **2019**, *28*, 1083-1094.
2. Brown, K.; Pompeo, F.; Dixon, S.; Mengin-Lecreulx, D.; Cambillau, C.; Bourne, Y. Crystal structure of the bifunctional n-acetylglucosamine 1-phosphate uridyltransferase from escherichia coli: A paradigm for the related pyrophosphorylase superfamily. *The EMBO journal* **1999**, *18*, 4096-4107.
3. Thoden, J.B.; Ruzicka, F.J.; Frey, P.A.; Rayment, I.; Holden, H.M. Structural analysis of the h166g site-directed mutant of galactose-1-phosphate uridylyltransferase complexed with either udp-glucose or udp-galactose: Detailed description of the nucleotide sugar binding site. *Biochemistry* **1997**, *36*, 1212-1222.
4. Ramakrishnan, B.; Qasba, P.K. Structure-based design of beta 1,4-galactosyltransferase i (beta 4gal-t1) with equally efficient n-acetylgalactosaminyltransferase activity: Point mutation broadens beta 4gal-t1 donor specificity. *The Journal of Biological Chemistry* **2002**, *277*, 20833-20839.
5. Pettersen, E.F.; Goddard, T.D.; Huang, C.C.; Couch, G.S.; Greenblatt, D.M.; Meng, E.C.; Ferrin, T.E. Ucsf chimera--a visualization system for exploratory research and analysis. *Journal of Computational Chemistry* **2004**, *25*, 1605-1612.
